# Length of Stay in Ambulatory Surgical Oncology Patients at High Risk for Sleep Apnea as Predicted by STOP-BANG Questionnaire

**DOI:** 10.1155/2016/9425936

**Published:** 2016-08-16

**Authors:** Diwakar D. Balachandran, Saadia A. Faiz, Mike Hernandez, Alicia M. Kowalski, Lara Bashoura, Farzin Goravanchi, Sujith V. Cherian, Elizabeth Rebello, Spencer S. Kee, Katy E. French

**Affiliations:** ^1^Department of Pulmonary Medicine, The University of Texas MD Anderson Cancer Center, Houston, TX, USA; ^2^Department of Biostatistics, The University of Texas MD Anderson Cancer Center, Houston, TX, USA; ^3^Department of Anesthesia and Perioperative Medicine, The University of Texas MD Anderson Cancer Center, Houston, TX, USA; ^4^Divisions of Pulmonary, Critical Care and Sleep Medicine, The University of Texas Health Science Center at Houston, Houston, TX, USA

## Abstract

*Background*. The STOP-BANG questionnaire has been used to identify surgical patients at risk for undiagnosed obstructive sleep apnea (OSA) by classifying patients as low risk (LR) if STOP-BANG score < 3 or high risk (HR) if STOP-BANG score ≥ 3. Few studies have examined whether postoperative complications are increased in HR patients and none have been described in oncologic patients.* Objective*. This retrospective study examined if HR patients experience increased complications evidenced by an increased length of stay (LOS) in the postanesthesia care unit (PACU).* Methods*. We retrospectively measured LOS and the frequency of oxygen desaturation (<93%) in cancer patients who were given the STOP-BANG questionnaire prior to cystoscopy for urologic disease in an ambulatory surgery center.* Results*. The majority of patients in our study were men (77.7%), over the age of 50 (90.1%), and had BMI < 30 kg/m^2^ (88.4%). STOP-BANG results were obtained on 404 patients. Cumulative incidence of the time to discharge between HR and the LR groups was plotted. By 8 hours, LR patients showed a higher cumulative probability of being discharged early (80% versus 74%, *P* = 0.008).* Conclusions*. Urologic oncology patients at HR for OSA based on the STOP-BANG questionnaire were less likely to be discharged early from the PACU compared to LR patients.

## 1. Introduction

Obstructive sleep apnea (OSA) is characterized by repetitive episodes of apnea or reduced inspiratory airflow due to upper airway obstruction, resulting in increased respiratory effort, oxyhemoglobin desaturation, and frequent neuronal arousals during sleep. General anesthesia and perioperative analgesia may often exacerbate this pathophysiology; thus, OSA patients may suffer from postoperative respiratory, cardiovascular, and neurological complications [1–6]. OSA can further complicate perioperative care by impacting recovery time and increasing hospital length of stay (LOS). Surprisingly, up to 70% of patients undergoing surgery may have undiagnosed OSA, thus increasing the need to develop strategies to identify patients at risk [7, 8].

The STOP-BANG is a simple patient-administered questionnaire developed by Chung and associates with screen for OSA in the perioperative setting, and it has a high sensitivity for detecting OSA in this setting [9]. The STOP-BANG consists of 8 items producing a numerical score that can be used to classify patients as low risk (LR, score < 3), intermediate risk (IR, score of 3 or 4), and high risk (HR, score ≥ 5) for OSA. Several studies have demonstrated that patients with higher preoperative STOP-BANG scores have increased postoperative complications, including increased difficulty with intubations and unanticipated critical care admissions [10–12].

Although the American Society of Anesthesiologists (ASA) and the Society for Ambulatory Anesthesia have issued guidelines for the perioperative management of OSA, standardized data addressing the appropriate duration and setting for monitoring patients undergoing ambulatory procedures is lacking [13–15]. Furthermore, the optimal management strategy for mitigating the risk of complications in patients with OSA remains unclear. No previous study has examined the incidence and risk of perioperative OSA in an oncologic population. The purpose of our study was to use the STOP-BANG questionnaire to classify patients as LR or HR for OSA and determine if HR patients were less likely to be discharged early from the PACU when compared to LR patients.

## 2. Methods

This study is a retrospective review of the medical records of 1666 consecutive patients with urologic cancer who underwent cystoscopy in the ambulatory surgical center at University of Texas MD Anderson Cancer Center in Houston, Texas, from March 2013 to February 2015. The study was conducted with the approval of our institution's institutional review board. Due to the retrospective nature of this study and adherence to the established standard of care, a waiver of informed consent was obtained.

Patients and clinical characteristics included basic demographics: (i) age, (ii) sex, and (iii) body mass index (BMI). The STOP-BANG questionnaire was administered to 404 patients as part of the standard of care while in the perioperative anesthesia assessment center to determine risk of OSA. The STOP-BANG survey consists of the following 8 items: (i) loud snoring; (ii) being tired, fatigued, or sleepy; (iii) observed stop of breathing; (iv) high blood pressure; (v) body mass index (BMI) > 35 kg/m^2^; (vi) age older than 50; (vii) neck size > 17 inches (40 cm); and (viii) male gender. Patients were assigned a value of “1” if the response was affirmative with each item and a “0” if negative. STOP-BANG scores range from 0 to 8. We utilized a dichotomous framework with HR patients defined as those with a STOP-Bang score ≥ 3 and LR patients defined as those with a STOP-Bang score < 3 [9]. Complete STOP-BANG scores were acquired on 404 patients. If a patient had more than one cystoscopy during the course of the study period, the STOP-BANG score and other parameters were gathered from the first procedure.

Medical information from the time spent in the postanesthesia care unit (PACU) was obtained from an integrated perioperative electronic medical record (PICIS, Westfield, MA). Parameters recorded while in the PACU included the following: (i) vital signs during observation, (ii) time of admission to PACU, (iii) time of discharge from PACU, and (iv) procedure time. Length of stay (LOS) was defined as the duration of time in PACU, and it was calculated as the time from admission to the PACU to the time the patient met established discharge criteria such as adequate pain control, voiding, and tolerance of oral intake. All patients were kept for a minimum of 2 hours in the PACU. Oxygen desaturation was defined as oxygen saturation less than 93% with or without supplemental oxygen for 10 seconds, as recorded by pulse oximetry.

### 2.1. Statistical Analysis

Descriptive statistics were used to summarize patients' demographic and clinical characteristics by LR and HR status established by the STOP-BANG questionnaire. Histograms, box plots, and one-way scatter plots were used to visually assess the distributions of continuous characteristics of interest. Independent samples* t*-tests, or Wilcoxon rank-sum tests if more appropriate, were used to compare continuous characteristics between the LR and HR patients. In cases where the data was categorical, comparisons were made using Chi-square tests. Our primary hypothesis was that HR patients would have longer discharge times relative to LR patients. Cumulative incidence plots were generated to illustrate the cumulative probability of patient discharge at any time during the postoperative follow-up period. The distributions of discharge times were compared between HR and LR patients using a log-rank test. Secondary aims included the assessment of LOS and more frequent episodes of oxygen desaturation where a correlation coefficient was estimated. All statistical tests were two-sided, and a *P* value < 0.05 was used to signify statistical significance. All analyses were performed using Stata version 12.0 (Stata, College Station, TX).

## 3. Results

Four hundred and four consecutive cancer patients provided complete information to obtain a STOP-BANG score. Patient characteristics are summarized in Table 1. The majority of patients surveyed were men (77.7%), over 50 years of age (90.1%), and had BMI < 30 kg/m^2^ (88.4%). Anesthesia duration between the two groups was similar with a mean of 63 minutes in the LR group and 61.3 minutes in the HR group (*P* = 0.560, Table 2).

**Figure 1:**
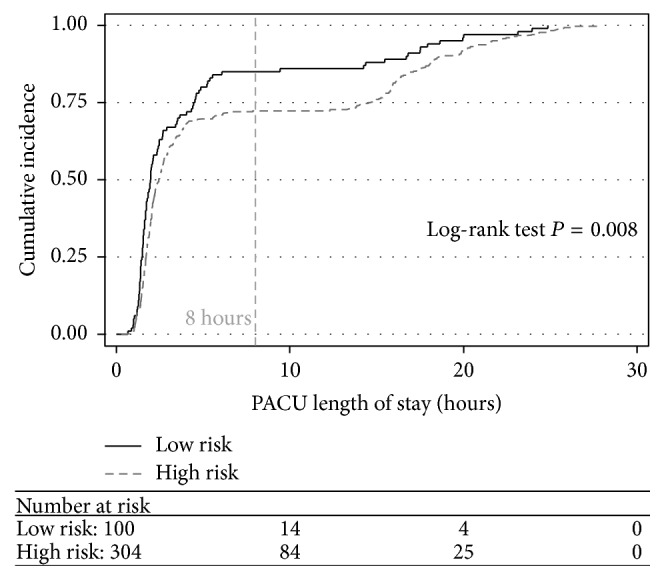
Cumulative incidence of discharge over time in the PACU.

**Figure 2:**
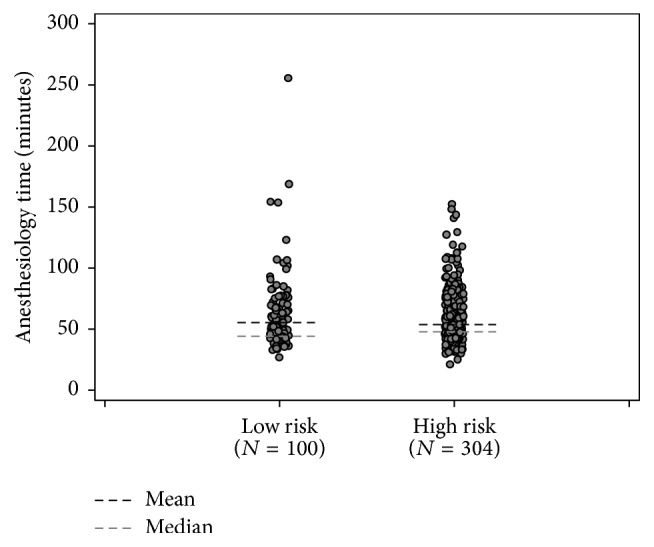
Procedure time by low and high risk patients based on STOP-BANG questionnaire.

The patients were categorized into HR and LR categories on the basis of the patients' STOP-BANG scores (Table 2). The average PACU LOS, procedure time, and frequency of oxygen saturation <93% during the PACU stay are also shown in Table 2 for both the HR and LR categories. Overall, the mean PACU LOS was 372.4 minutes.  Figure 1 shows the cumulative incidence plots which illustrate the time to discharge between HR and the LR groups. By 8 hours, LR patients showed a higher cumulative probability of being discharged early (80% versus 74%, *P* = 0.008). The separation of the curves does not persist beyond 20 hours, although, by then, most patients were discharged. All patients were discharged home, and there were no patients admitted overnight.  Figure 2 demonstrates that the mean duration of anesthesia in LR and HR patients was similar.

**Figure 3:**
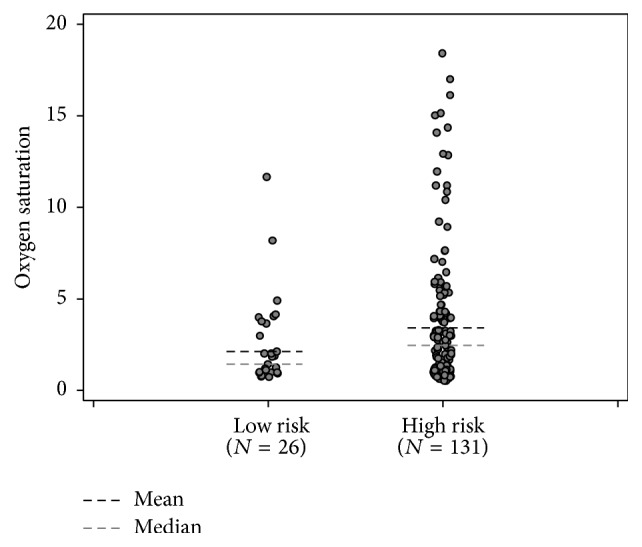
Oxygen saturation by low and high risk patients based on STOP-BANG questionnaire.

Oxygen desaturation was examined as a potential etiology for increased LOS (Figure 3), but there was no statistical difference in the frequency of oxygen desaturation between the LR and HR groups (*P* = 0.131). However, there was a statistically significant positive correlation between LOS and frequency of oxygen desaturation (*r* = 0.30, *P* < 0.001).

## 4. Discussion

Our study demonstrates a longer LOS for those stratified as HR patients based on the STOP-BANG questionnaire in an ambulatory oncologic setting. Although more frequent oxygen desaturations were not observed, an increased time to discharge between the LR and HR groups was noted. Our data further corroborates other published works using this screening tool for postoperative outcome in general surgery patients. It is the first study to utilize the STOP-BANG and assess outcomes in a purely oncologic and ambulatory population.

Many oncologic patients require general anesthesia and surgery multiple times throughout the treatment of their cancer. Although little is known about the clinical impact of OSA in surgical oncology patients, undiagnosed OSA in the perioperative setting has been shown to have more complications, especially cardiac or respiratory in origin [16]. The nature of anesthesia places patients with sleep apnea at higher risk due to multiple factors including medications (sedation, analgesics), alterations in upper airway patency, and positioning [17]. The supine position required in many surgeries may create dead space thus reducing lung volumes and oxygen saturations. Atelectasis as a result of pain induced splinting or sedation may further contribute to hypoventilation and respiratory insufficiency [18]. Sedation, neuromuscular blockade, and postsurgical opioid pain medications decrease arousal and may elicit sleep apnea symptoms postoperatively by decreasing neuromuscular tone in the upper airway [19, 20]. Finally, sleep fragmentation after surgery can reduce rapid eye movement (REM) sleep stage, immediately postoperatively, following which REM sleep rebound can occur. REM sleep rebound is associated with muscle atonia and may promote sleep apnea and cardiac dysrhythmia days after surgery [7, 21]. We did not observe any new onset atrial fibrillation, ICU admissions, or respiratory distress in our cohort, and we suspect the selection of an ambulatory population with short procedure times likely influenced the lack of these complications. In general, however, complications related to OSA in the perioperative setting may result in longer length of stay and consequent increased resource utilization.

The application of the STOP-BANG questionnaire in an oncologic setting is novel. Intuitively, one may question the use of this tool since most assume that cancer patients will be fatigued or tired, thus potentially reducing the specificity of the survey. Interestingly, in our study, only 43.8% of cancer patients affirmed feeling “tired.” It is unclear if that fatigue is due to undiagnosed OSA or cancer, but regardless the nonuniform response shows promise for this tool in surgical cancer patients. Given the success of this tool in the perioperative setting, the application to our cancer population seems logical. It is important to recognize however that the use of the STOP-BANG or any screening tools to diagnose sleep apnea and predict outcomes has limitations, for OSA shares common symptoms with many other diseases, thus lowering their specificity [22]. The score, itself, contains elements such as obesity, age, hypertension, gender, and fatigue. Each of these factors might be associated with increased postoperative stay, regardless of the presence or absence of OSA. Disentangling these contributors to sleep apnea from the disease itself, therefore, is challenging [23]. Finally, the discovery of an early discharge in LR patients despite short procedure duration in an outpatient setting further demonstrates the potential robustness of the STOP-BANG questionnaire. Our study establishes areas of application for the STOP-BANG questionnaire in cancer patients.

Our study has limitations inherent to most retrospective studies. We tried to use an adequate sample size and had controls for many of the variables. An electronic medical record and an integrated perioperative medical record were used so loss of patient data was marginal. The procedures for each patient, including the anesthetic agents, process of care, monitoring, and standardized criteria for discharge from the PACU, were the same. Although discharge criteria were standardized, variability due to factors, related to the delivery of care and unrelated to the risk for sleep apnea, such as patient transportation needs, nursing shift changes, and provider implementation, may have affected LOS. We attempted to minimize this variability by evaluating a large number of consecutive patients. All our patients were ambulatory and procedure times were similar and brief, so there may be selection bias since these procedures were not extensive or prolonged. In our study, the difference in time to discharge did not persist beyond 20 hours, although, by then, most patients were discharged. This finding may signify that factors other than risk for sleep apnea may be responsible for prolonged time to discharge from the PACU for some patients. Expansion of our study to include the nonambulatory setting and longer surgery times may identify patients at increased risk of respiratory or cardiovascular complications. Evaluation of these patients may also magnify the difference found between the HR and LR groups.

Our findings support the recommendations of the ASA guidelines, which call for more intensive monitoring of patients with OSA [13]. Further delineation of the patient population and surgical procedures which are less prone to complications and lower risk, respectively, may help guide escalation of care for OSA. This would be especially beneficial for anesthesiologists, given the advent of the Affordable Care Act. As in other medical specialties, anesthesiologists will be asked to focus on cost containment by increasing the number of procedures performed in the ambulatory setting, without compromising patient safety. Alternatively, monitoring for complications beyond the PACU may also be considered, for it is known that the effects of anesthesia on respiratory and sleep patterns reach their peak approximately 72 hours postoperatively [24]. Further prospective studies in the oncologic population undergoing longer and more complex surgeries in hospitalized patients with the STOP-BANG score could also better define the role of this screening tool and correlate with more intensive and prolonged monitoring.

To date, this study represents the largest group of cancer patients screened preoperatively with the validated STOP-BANG. This study demonstrates that cancer patients undergoing a relatively minor procedure with less known cardiorespiratory morbidity may benefit from screening for OSA with the STOP-BANG questionnaire. Knowing the average duration of LOS for HR patients may assist healthcare providers in selecting an appropriate duration of monitoring. If the LOS is higher for ambulatory patients with suspected OSA, then guidelines that recommend an increase in the duration of observation are justifiable and worth the increased resources that such monitoring entails. Systematically designed studies to better define which patient may undergo higher risk surgery in ambulatory centers safely are needed. The potential role for the STOP-BANG survey in cancer patients may be greater than previously anticipated.

## Figures and Tables

**Table 1:** Patient characteristics.

	*N* = 404^*∗*^	Percentage
BMI (kg/m^2^)		
Mean ± SD	28.5 ± 5.7	
Median (min to max)	27.3 (16.5 to 51)	

Neck circumference (cm)		
Mean ± SD	39.8 ± 4.6	
Median (min to max)	40 (28 to 54)	

PACU LOS (minutes)		
Mean ± SD	372.4 ± 433.0	
Median (min to max)	137.5 (39 to 1667)	

Procedure time (minutes)		
Mean ± SD	61.7 ± 24.2	
Median (min to max)	55 (26 to 256)	

Oxygen saturation below 93% (count)		
Mean ± SD	3.7 ± 3.8	
Median (min to max)	2 (1 to 18)	

STOP-BANG items		
Snoring		
No	305	75.5
Yes	99	24.5
Tiredness		
No	227	56.2
Yes	177	43.8
Observed apneas		
No	361	89.4
Yes	43	10.6
High blood pressure		
No	169	41.8
Yes	235	58.2
BMI > 35 kg/m^2^		
No	357	88.4
Yes	47	11.6
Age > 50 years		
No	40	9.9
Yes	364	90.1
Neck > 40 cm		
No	234	57.9
Yes	170	42.1
Gender		
Female	90	22.3
Male	314	77.7

STOP-BANG criteria		
Low risk (<3)	100	24.8
High risk (≥3)	304	75.2

Bicarbonate ≥ 28 mmol/L		
No	194	48
Yes	210	52

BMI, body mass index; SD, standard deviation; PACU, postanesthesia care unit. ^*∗*^404 patients provided complete STOP-BANG information to derive a total score.

**Table 2:** Patient characteristics based on STOP-BANG questionnaire.

	STOP-BANG		
	STOP-BANG < 3 (LR) *N* = 100	STOP-BANG ≥ 3 (HR) *N* = 304	*P* value
BMI (kg/m^2^)			
Mean ± SD	25.7 ± 3.9	29.4 ± 5.9	—
Median (min to max)	25.4 (18.4 to 36.8)	28 (16.5 to 51)	

Neck circumference (cm)			
Mean ± SD	36.0 ± 3.3	41.1 ± 4.2	—
Median (min to max)	36 (28 to 46)	41 (29 to 54)	

PACU LOS (minutes)			
Mean ± SD	278.6 ± 358.6	403.2 ± 451.1	—^#^
Median (min to max)	117.5 (39 to 1492)	146 (44 to 1667)	

Anesthesia duration (minutes)			
Mean ± SD	63.0 ± 32.1	61.3 ± 20.9	0.560^*∗*^
Median (min to max)	52 (30 to 256)	55.5 (26 to 147)	0.277^*∗∗*^

Oxygen sat below 93% (count)			
Mean ± SD	2.7 ± 2.6	3.9 ± 3.9	0.131^*∗*^
Median (min to max)	2 (1 to 12)	3 (1 to 18)	

LR, low risk; HR, high risk; SD, standard deviation.

—: BMI and neck circumference were used to create the STOP-BANG score.

^#^ *P* value is not provided; PACU LOS has a bimodal distribution.

^*∗*^ *P* value is based on independent samples *t*-test.

^*∗∗*^ *P* value is based on Wilcoxon rank-sum test.
